# Precision Immunotherapeutics for Glioblastoma: Current Approaches and Emerging Strategies in 2026

**DOI:** 10.3390/cells15060561

**Published:** 2026-03-20

**Authors:** James Poe, Claire Kim, Campbell Coleman, Hieu Nguyen, Vaithish Velazhahan, Brandon Bergsneider, Vivek Sanker, Samuel Kim, Yijiang Chen, Matthew Abikenari, John Choi, Michael Lim

**Affiliations:** 1 Department of Neurosurgery, School of Medicine, Stanford University, Stanford, CA 94305, USA; jamespoe@stanford.edu (J.P.); nguyenhm@stanford.edu (H.N.); mattabi@stanford.edu (M.A.);; 2 Department of Radiation Oncology, School of Medicine, Stanford University, Stanford, CA 94305, USA

**Keywords:** glioblastoma, tumor microenvironment, precision immunotherapy, T cell exhaustion, metabolic reprogramming, checkpoint immunotherapy, interferon signaling, spatial immune organization

## Abstract

Glioblastoma (GBM) persists as one of the greatest challenges in the treatment of human cancer, despite extensive efforts to leverage the therapeutic potential of immunotherapy. While checkpoint blockade and other forms of immunotherapy have revolutionized the treatment of various cancers, their therapeutic efficacy in GBM has been hindered by the profound immunosuppressive environment, spatial heterogeneity, and dynamic immune metabolic challenges associated with the tumor microenvironment. In this review, we will synthesize recent advances and insights to develop a next-generation framework for GBM immunotherapy based on systems biology approaches to understanding the complex interplay between GBM and the immune system, as opposed to single-axis approaches to immune activation and modulation. We will discuss how the functional competence of the interferon system, myeloid antigen presentation status, T-cell clone status, spatial organization of the immune microenvironment, and resource competition between GBM and the immune system dictate therapeutic responsiveness. Furthermore, the current paper elucidates how recent advances in spatial transcriptomics, single-cell analysis, and high-parameter imaging enable us to understand how immune phenotype status varies across GBM regions and treatment status, and how this information can be used to develop predictive and pharmacodynamic biomarkers of therapeutic efficacy and failure. We will then discuss how these advances form the basis for rational combination approaches to GBM immunotherapy, which involve the integration of checkpoint blockade with metabolic reprogramming, myeloid modulation, and interferon system reactivation, and how artificial intelligence-based analytics and adaptive clinical trial design can guide the development of biomarker-based therapeutic selection approaches.

## 1. Introduction

Glioblastoma (GBM) is the most prevalent and aggressive primary malignant brain tumor in adults, accounting for about 52.2% of all primary brain malignancies. The estimated incidence of GBM is 3.28 cases per 100,000 individuals, with a median age at diagnosis of 66 years [1]. The clinical burden of GBM extends beyond tumor location-related adverse effects, as GBM not only causes widespread metabolic derangements, but it is inherently a diffusely infiltrative disease that can disrupt synaptic function, white matter tracts, and immune function, effecting a major decline in the quality of life of patients [2]. Despite aggressive treatment strategies, which typically involve a combination of maximal surgical resection with radiotherapy and adjuvant temozolomide, median overall survival (OS) remains only around 12–18 months [1,3]. Furthermore, GBM is associated with high recurrence rates, largely attributable to tumor heterogeneity and inherent resistance mechanisms [4].

Immunotherapy has transformed therapeutic outcomes in several cancers, including melanoma, lung, hematologic, breast, and renal cancer, where significant increases in overall survival have been demonstrated [5,6,7,8]. However, these successes have not been replicated in GBM, where immunotherapies have largely failed to elicit meaningful clinical outcomes [9]. Factors contributing to the suboptimal therapeutic responses include a profoundly immunosuppressive tumor microenvironment (TME) [10], the presence of glioblastoma stem cells that evade immune recognition [11], and adaptive resistance mechanisms that undermines the efficacy of immune checkpoint inhibitors [4].

Our review aims to bridge the gap between the clinical failures in GBM treatment and emerging mechanistic insights to inform the development of innovative therapeutic strategies. We underscore the importance of integrating tumor metabolism, the impact of the immunosuppressive TME, and the latest advances in immune engineering to expand the scope of immunotherapeutic approaches. The rich heterogeneity of GBM, characterized by its cellular diversity, necessitates the exploration of combinatorial therapies tailored to the unique characteristics of each patient’s tumor. Our review addresses the essential need for a paradigm shift in GBM treatment strategies, emphasizing the contemporary utilization of multi-omic technologies to design rational precision medicine immunotherapy trials based on patient-specific biomarkers and novel immunotherapeutic strategies.

## 2. The Unique Immunobiology of GBM

### 2.1. Immunologically Cold Tumor Hallmarks

GBM is classically associated with poor immunogenicity [12] and a “cold” tumor phenotype, characterized by a low tumor mutational burden (TMB) [13]. Genomic analyses indicate that GBM harbors markedly fewer somatic mutations than most other solid malignancies, with median TMB levels of ~1–3 mutations/Mb [14,15,16], substantially lower than those observed in highly immunogenic tumors such as melanoma (~10–100 mutations/Mb) and non-small cell lung cancer (~6–12 mutations/Mb) [17,18,19,20,21]. The paucity of mutations in GBM limits the identification of tumor-specific neoantigens capable of eliciting effective T-cell responses [22], and, in turn, reduces the efficacy of immune checkpoint inhibitors and related immunotherapies that rely on robust antigen presentation and recognition [23]. Further, the progressive loss of immunogenic neoantigens due to immune selection leads to insufficient antitumor immune activation and facilitates persistent immune escape in GBM [24].

Tumors further evade immune surveillance through downregulation of major histocompatibility complex (MHC) class I expression, diminishing presentation of neoantigens to CD8^+^ cytotoxic T cells [25]. In GBM, this downregulation is observed notably at the invasive edge of the tumor compared with the tumor core, allowing glioma cells to invade the surrounding brain tissue while escaping detection by cytotoxic T cells [26]. The loss of MHC-I expression has been correlated with poorer prognoses and response to immune checkpoint inhibitors in several cancer patient cohorts and clinical trials [27], and recent pan-cancer analyses indicate that higher MHC-I expression correlates with improved response to PD-1/PD-L1 inhibitors [28].

### 2.2. Tumor Microenvironment (TME) Barriers

The GBM TME is profoundly immunosuppressive, inhibiting anti-tumor immune responses through a multilayered network of inhibitory pathways [27,29,30]. For instance, canonical immunosuppressive checkpoints such as programmed death-ligand 1 (PD-L1) and cytotoxic T-lymphocyte-associated protein 4 (CTLA-4) are overexpressed within the GBM TME and promote functional exhaustion of T cells [31,32,33,34,35]. PD-L1, expressed on both tumor cells and infiltrating myeloid populations including tumor-associated macrophages (TAMs), binds inhibitory PD-1 receptors on activated T cells, suppressing their cytotoxic function [36,37]. Concurrently, CTLA-4 skews the local immune balance toward regulatory T cell (Treg) dominance. Tregs express high levels of CTLA-4, enabling them to remove co-stimulatory ligands CD80 and CD86 from antigen-presenting cells, blocking CD28-mediated activation of effector T cells [38,39,40,41]. While PD-1 and CTLA-4 are well-studied, recent studies show that GBM-infiltrating T cells also commonly express high levels of other checkpoint receptors, including T-cell immunoglobulin and mucin-domain-containing-3 (TIM-3), lymphocyte activation gene-3 (LAG-3), and T-cell immunoreceptor with Ig and ITIM domains (TIGIT) [42,43,44,45,46].

Additionally, the TME further suppresses T cells and NK lymphocytes through immunosuppressive factors secreted by tumor-associated macrophages (TAMs), myeloid-derived suppressor cells (MDSCs), and neutrophils [47,48], including arginase-1 (Arg-1) [49,50,51,52,53], inducible nitric oxide synthase (iNOS) [54,55,56,57,58], reactive oxygen species (ROS) [54,58,59,60,61,62], and anti-inflammatory cytokines like TGF-β and IL-10 [63,64,65], which inhibit T cell activity and promote Treg expansion.

### 2.3. Physical Barriers

GBM is further protected by physical and metabolic barriers that collectively limit immune surveillance. Central among these is the blood–brain barrier (BBB), which tightly regulates the passage of molecules and cells from systemic circulation into the brain parenchyma [66]. In GBM, tumor-driven angiogenesis disrupts the BBB and gives rise to the blood–tumor barrier (BTB), which exhibits increased but heterogeneous permeability [67,68,69]. While the BTB is more permeable than the normal BBB in the tumor core, it remains intact or only partially compromised at the invasive tumor margins, limiting delivery of chemotherapeutic agents and immune effector cells to infiltrative tumor regions [70,71].

Hypoxia is another defining physical feature of the GBM microenvironment, resulting from rapid tumor growth, aberrant vasculature, and microvascular thrombosis [72,73]. Hypoxic conditions stabilize hypoxia-inducible factors (HIFs), which drive angiogenesis, promote glioma stem cell maintenance, and shift tumor cell metabolism from oxygen-dependent to oxygen-independent pathways.

Metabolic competition within the GBM microenvironment further complicates immune response. The preferential reliance of GBM cells on aerobic glycolysis (the Warburg effect) to meet high energy demands leads to lactate accumulation, acidifying the local environment and favoring M2-like TAM polarization and Treg-mediated suppression of effector T cell function [74,75,76,77,78]. Additionally, GBM cells upregulate the tryptophan-kynurenine pathway through increased expression of indoleamine 2,3-dioxygenase (IDO) and tryptophan 2,3-dioxygenase (TDO), resulting in tryptophan deletion and accumulation of immunosuppressive kynurenine metabolites, which inhibit T cell proliferation and promote regulatory T cell differentiation [79,80,81].

Another key reason immunotherapy has failed to deliver for GBM is that the immune bottleneck is typically initiated before T cell effector function, at the myeloid cell dominance and defective antigen presentation stages. GBM tumors are heavily infiltrated with resident microglia, macrophages, and MDSCs that form suppressive microenvironments through checkpoint ligand expression, suppressive cytokines, and defective co-stimulation. In effect, it means that even when present, T cells are typically primed suboptimally, mislocalized, or continually suppressed by myeloid-derived signals. The consequence is that there is a hard cap on the potential benefit that can be achieved with PD-1 and CTLA-4 inhibition. By including this early on, it becomes clear that failed immunotherapy in GBM is not due to a lack of immune activation, but myeloid cell-driven immune suppression [55,73,79,82].

Hence, in addition to immune cell composition, GBM tumors are characterized by a restrictive metabolism characterized by hypoxia, nutrient limitation, lactic acidosis, and adenosine signaling that directly impacts immune fitness. In particular, hypoxia drives HIF-dependent pathways that are known to support angiogenesis and stemness while concurrently directing infiltrating myeloid cells toward suppressive polarization and limiting lymphocyte effector cell persistence. In parallel, defective T cell proliferation and cytokine production are driven by GBM-driven glycolysis and acidity, while simultaneously reinforcing tolerogenic antigen-presenting cell states and defective immune suppressive pathways such as IDO/TDO/kynurenines-AhR. In such a microenvironment, immune checkpoint therapy is initiated with a backdrop where the energy status and redox status are not conducive to cytotoxic T cell function [73,80,83].

### 2.4. Emergent Concepts

In recent years, a growing body of evidence has reshaped our understanding of the GBM TME, shifting the view towards a complex “tumor–stroma–immune” ecosystem. Stromal cells such as astrocytes and cancer-associated fibroblasts (CAFs) emerge not just as bystanders but as active architects of immunosuppression and tumor progression [84,85,86,87,88,89,90]. CAFs secrete immunosuppressive cytokines (e.g., TGF-β, IL-6), chemokines (e.g., CXCL12, CCL2), and metabolites (e.g., PGE2), which recruit regulatory immune cells such as Tregs and MDSCs, inhibit lymphocyte function, and upregulate immune checkpoints (such as PD-1/PD-L1, CTLA4) on both stromal and immune cells [91,92,93]. CAFs also remodel the extracellular matrix, creating physical barriers to immune cell infiltration and facilitating therapy resistance [94,95]. Recent spatial and single-cell analyses reveal that close spatial interactions between CAFs and tumor cells also further intensify immunosuppression, with mixed distributions of CAFs and cancer cells correlating with poor response to immunotherapy and reduced CD8+ T cell infiltration [96].

Astrocytes are similarly active modulators of the GBM microenvironment. They undergo transcriptional and functional reprogramming by GBM cells to adopt immunosuppressive phenotypes. Tumor-associated astrocytes secrete immunosuppressive cytokines (TGF-β, IL-10, G-CSF), recruit and polarize myeloid cells, and directly suppress T cell function by inducing T cell apoptosis via TRAIL, a process driven by tumor-derived IL-11 and STAT3 signaling [89,97,98].

Single-cell and spatial transcriptomic studies reveal distinct astrocyte subpopulations with specialized roles in various tumor regions that influence local immune cell infiltration and activity [99]. Bidirectional signaling pathways such as ANXA1-FPR1 limit pro-inflammatory responses and immunogenic cell death [100], and astrocytes also provide metabolic support to tumor cells via cholesterol provision [98]. Through crosstalk with other glial cells such as microglia, astrocytes act as pivotal regulators of GBM immune landscapes. For instance, Heiland et al. demonstrated that astrocytes only secrete anti-inflammatory cytokines like IL-10 and TGF-β in the presence of microglia or macrophages [97,101]. These findings highlight the potential of astrocytes as targets to disrupt tumor–stroma–immune networks and enhance immunotherapeutic efficacy.

Together, these findings underscore that GBM immunobiology extends beyond tumor-intrinsic mechanisms, encompassing a dynamic tumor–stroma–immune ecosystem in which astrocytes, CAFs, and other stromal elements actively shape immune suppression, metabolic support, and therapy resistance. Targeting these stromal–immune interactions offers a promising avenue to remodel the TME and enhance the efficacy of immunotherapeutic strategies. Table 1 summarizes the principal mechanistic axes of immunotherapy resistance in glioblastoma, integrating tumor-intrinsic, myeloid, metabolic, and spatial determinants with corresponding biomarker readouts and therapeutic leverage points.

## 3. Clinical Landscape of Immunotherapy in GBM

Immunotherapy has evolved in the past years, but its success in treating GBM has been limited due to the GBM microenvironment’s suppression of effective anti-tumor immune responses.

### 3.1. Immune Checkpoint Inhibitors (ICIs)

Immune checkpoint inhibitors (ICIs) have been studied in multiple randomized trials for GBM. In newly diagnosed MGMT-unmethylated GBM, the phase III CheckMate 498 study compared radiation therapy and adjuvant nivolumab, a PD-1 inhibitor, (RT + NIVO) to the standard-of-care of radiation therapy and temozolomide (RT + TMZ) and demonstrated that RT + NIVO actually had lower median overall survival compared to RT + TMZ. Similarly, the CheckMate 548 trial compared RT + TMZ combined with NIVO to RT + TMZ combined with placebo in newly diagnosed MGMT-methylated GBM patients and showed no differences in overall or progression-free survival. In recurrent GBM, the phase III CheckMate 143 trial compared nivolumab with bevacizumab and found that nivolumab did not improve overall survival compared to bevacizumab [102]. This lack of response to PD-1 inhibitors in recurrent GBM appears to be independent of whether the inhibitor is given neoadjuvantly or adjuvantly, as a recent pilot trial in surgically resectable, recurrent GBM (NCT02852655) demonstrated that neoadjuvant administration of pembrolizumab, another PD-1 inhibitor, prior to surgery did not improve outcomes compared to adjuvant pembrolizumab alone [82].

PD-L1 inhibitors, such as durvalumab and atezolizumab, have also been investigated in single-arm phase I and II trials that have shown survival comparable to, but not exceeding, that of historical controls treated with standard-of-care [103,104]. Multiple other trials studying various combinations of checkpoint inhibitors such as PD-1, PD-L1, CTLA4, and LAG3 inhibitors are also ongoing, but none to date have yet demonstrated improved survival compared to standard-of-care [105].

### 3.2. Cancer Vaccines

Cancer vaccines aim to stimulate T cells to recognize neoantigens in GBM and include dendritic cell vaccines (DCVs), peptide vaccines, mRNA vaccines, and viral vector vaccines [106]. Dendritic cell vaccines (DCVs) have been shown to effectively induce anti-tumor immune responses in certain patients, and these responses may be further enhanced when combined with chemo- or radiotherapy [107]. For example, in a recent phase 3 prospective externally controlled cohort trial, DCVax-L improved overall survival and increased the long-term tails of the survival curves in patients with both newly diagnosed and recurrent GBM when compared to contemporaneous, matched external control patients treated with standard-of-care [108]. Rindopepimut, an EGFRvIII peptide vaccine, was studied in the phase III ACT IV trial but did not increase survival in newly diagnosed EGFRvIII-positive GBM [109]. Personalized, mutation-specific neoantigen vaccines were able to induce strong immune responses and T-cell infiltration, despite GBM’s “cold” microenvironment [110,111]. However, the long-term benefit and durability of neoantigen vaccines is not definitive.

### 3.3. Oncolytic Viruses

Oncolytic virotherapy aims to lyse cancer cells and use the resulting debris to induce a secondary anti-tumor immune response [112]. A phase 1 trial of the oncolytic adenovirus Delta24-RGD (DNX-2401) in patients with recurrent GBM demonstrated anti-tumoral immune response in multiple patients and complete regression of tumor in one patient [113]. DNX-2401 has a mutation in the E1A gene, which prevents it from binding the retinoblastoma (Rb) protein, thus restricting its replication to cancer cells that often have defective Rb signaling, while leaving normal cells unaffected [114]. PVSRIPO, a poliovirus-based vaccine, targets CD155 expression, which is often upregulated in tumor cells, in order to replicate itself [115]. A non-randomized early-phase trial found that PVSRIPO improved survival rates in recurrent GBM patients compared to historical controls, but its long-term benefit is still being studied [116]. HSV-G47Δ (Delytact), a herpes simplex virus type 1, has deletions in ICP6, γ34.5, and α47, which restricts viral replication to cancer cells, and in doing so, dysregulates non-coding RNAs to promote cytotoxicity [117]. In a phase 2 trial, patients with residual or recurrent GBM who received G47/if aaΔ treatment demonstrated T-cell infiltration and both increased overall and progression-free survival. This led to a conditional, time-limited approval of G47Δ for glioma patients in Japan [118].

### 3.4. Cell-Based Therapies

Cell-based therapies have also been explored in the context of glioblastoma [119,120,121,122]. CAR T cells have been designed to target cancer-specific mutations and antigens upregulated in tumors cells like EGFRvIII, IL13Rα2, HER2, and GD2 [121,123]. An in-human trial found that infusing CAR T cells intravenously in patients with recurrent GBM to target EGFRvIII was safe and promoted T-cell infiltration [124]. Similarly, HER2-CAR T cells demonstrated strong anti-tumor immune responses in vitro and inhibited tumor growth in vivo [125]. More recently, DNA-encoded tri-specific T-cell engagers (DTriTEs) were found to successfully bind to cancer cells that express EGFRvIII and IL13Rα2 to promote cytokine production and improve long-term survival in syngeneic mouse models with GBM tumor heterogeneity [126].

T cell receptor-engineered T cells (TCR-T) are another form of cell-based therapy being studied in GBM, as they have higher antigen sensitivity than CAR T cells and thus offer a wider range of antigens that they can recognize and target [127]. By targeting antigens like PTPRZ1, TCR-T cells can promote antigen-specific inflammatory signaling and anti-tumor immune activity [127].

### 3.5. Immunometabolic Crosstalk

Altered metabolic activity in GBM drives its profoundly immunosuppressive tumor microenvironment. GBM is highly aggressive and proliferates more rapidly than new vasculature can form, resulting in extensive intratumoral hypoxia [128]. Hypoxia stabilizes hypoxia-inducible factor-1α (HIF-1α), which induces expression of glycolytic enzymes and glucose transporters, enforcing a Warburg-like metabolic state [129]. HIF-1α signaling also promotes IL-8 production, which suppresses local T-cell activity and recruits TAMs [130,131]. In the setting of chronic hypoxia and tumor-derived cytokines, sustained HIF-1α signaling further skews TAMs toward an anti-inflammatory, M2-like phenotype [132].

During early antitumor immune responses, infiltrating T cells and NK cells produce interferon-γ (IFN-γ), which induces expression of indoleamine 2,3-dioxygenase (IDO) in tumor cells and TAMs [133,134,135]. IDO catalyzes the conversion of tryptophan to kynurenine, which is imported into T cells via the LAT1 transporter and activates the aryl hydrocarbon receptor (AhR) [136]. AhR signaling promotes FoxP3 expression and drives differentiation toward regulatory T cells, reinforcing immune suppression and enabling tumor immune evasion [136].

### 3.6. METRNL as a Reversible Immunometabolic Checkpoint in GBM

Recently, it has been proposed that the hypofunction of T cells in the context of GBM could be mediated not only by the action of the classical inhibitory receptors, but also by the effects of cytokines, leading to bioenergetic collapse. A recent study that analyzed multiple tumor types, including previously untreated glioblastoma, identified Meteorin-like (METRNL) as a feature that is conserved in checkpoint-high, antigen-experienced CD8+ TILs compared to patient-matched peripheral CD8+ populations [137]. Importantly, the function of METRNL has been characterized, demonstrating that it acts as an immunosuppressive signal under repeated tonic stimulation, where the presence of METRNL leads to decreased function and viability of CD8+ T cells, indicating that bioenergetic collapse is not merely a correlate but a cause of exhaustion-like failure states.

The mechanism by which METRNL leads to the failure of the function of T cells has also been characterized, demonstrating that it leads to mitochondrial depolarization, decreased oxidative phosphorylation, decreased mitochondrial reserve capacity, and increased glycolytic rate, the latter of which is not an adaptive process in the context of the GBM microenvironment, where energy is scarce and hypoxia is present. The implications of these findings for the treatment of glioblastoma are that, even in the context where checkpoint inhibitors partially restore the function of T cells, they could still be unable to carry out the function of killing tumor cells if the mitochondrial function has been actively impaired by the presence of the METRNL signal. Consistent with these findings, the knockdown of METRNL has been shown to improve the metabolic fitness of CD+ T cells, leading to improved tumor control in vivo, suggesting that the METRNL-E2F-PPARδ axis could act as a plausible and targetable ‘metabolic checkpoint’ that could be used in combination with other therapies to improve the bioenergetic function of T cells.

Lastly, a broader implication of this is that pushing glycolysis is not inherently immunostimulatory in GBM and may be protumoral depending on which immune compartments are being favored metabolically. There is increasing evidence that, in some contexts, regulatory immune compartments may be able to thrive under glycolysis-favoring conditions, while exhausted effector T cells do not. There is a perverse selection pressure here that means that metabolic states that favor glycolysis may drive or support the proliferation of suppressive immune states (such as Tregs), potentially exacerbating the immune problem that is being targeted by checkpoint inhibitors [137,138].

## 4. Practical Opportunity: Remodeling the TME Through the Restoration of Oxidative Fitness and Alleviation of Hypoxia

These considerations collectively point toward a design principle for the next generation of immunotherapy in GBM: a likely requirement for success is the remodeling of the tumor’s microenvironment such that the immune effectors are able to sustain oxidative fitness and evade the suppressive effects of hypoxia. Indeed, the therapeutics may encompass several approaches aimed at alleviating the tumor’s hypoxia and remodeling the vasculature, reducing the acidity and the activity of the lactate pathway, and directly supporting the mitochondrial health and oxidative phosphorylation capacity of the antitumor immune effectors. The objective is not just to add immunotherapy but to alter the conditions under which the immune response operates: from a state in which the glycolytic pathway is fixed and the immune response is suppressed by the tumor’s hypoxia to a state in which interferon responses, antigen presentation, and T cell-mediated cytotoxicity are energetically and spatially plausible. Such an approach is also well-aligned with the idea of biomarker-stratified trial design, in which the effect of the immunotherapy is verified through the observation of pharmacodynamic markers of microenvironment remodeling.

### Spatial Biology

Experimental and computational approaches that preserve the spatial context of cells, known as spatial biology methods, have substantially advanced our understanding of GBM heterogeneity by resolving spatial and metabolic relationships at single-cell resolution within intact tissue. For example, spatial transcriptomic studies demonstrate that TAMs localized to necrotic regions upregulate immunosuppressive and metabolically active markers such as ARG1, PD-L1, and CD39, consistent with a polarized, pro-tumor phenotype. In contrast, perivascular macrophages residing in relatively oxygenated niches often exhibit partial antigen-presentation signatures, including MHC class II expression and CIITA, reflecting preserved immune surveillance capacity rather than effective T-cell priming [137,138]. Spatial organization of T cells is similarly nonrandom, with exhausted CD8^+^ T cells preferentially clustering within nutrient-depleted and hypoxic regions [139].

**Figure 1:**
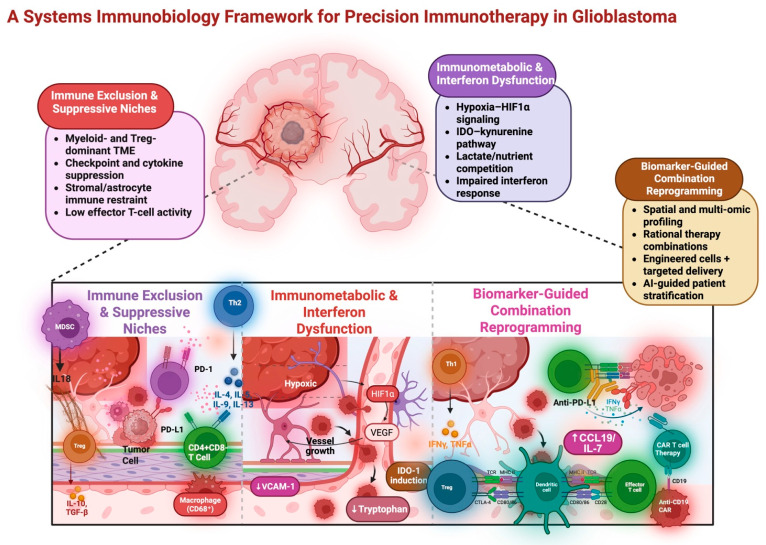
A systems immunobiology framework for precision immunotherapy in glioblastoma (GBM). This schematic illustrates the multi-level basis of immunotherapy resistance in GBM and proposes a biomarker-based approach to rational combination immunotherapy. The basis of GBM immunotherapy response is dictated by spatial, cellular, metabolic, and interferon-based interrelated programs rather than by an individual immune response. **Left panel: Immune exclusion and suppressive niches:** The GBM tumor microenvironment (TME) is often dominated by suppressive myeloid cells and Tregs, with high checkpoint and inhibitory cytokine signaling that limits effector T-cell functions. The stroma and astrocyte-derived signals further contribute to immune suppressive microenvironments, which limit CD8+ T-cell infiltration and functions. **Middle panel: Immunometabolic and interferon dysfunction:** Hypoxia and HIF1α-based programs, aberrant angiogenic signaling, and competitive metabolic pressure shape immune cell fitness and phenotype. IDO-kynurenine pathway activation and nutrient depletion drive Treg-based suppressive responses and T-cell dysfunction, while dysregulated interferon responses impair antigen presentation and immune priming. **Right panel: Biomarker-guided combination reprogramming:** Spatial and multi-omics-based approaches identify tumor immune-metabolic states that can be targeted by combination immunotherapies using rational approaches to combination therapy. Biomarker-based stratification supports adaptive and precision-based approaches to immunotherapy selection and trial design. Created in Biorender. Abikenari. (2026).

By integrating spatial cell density with co-expression of receptors and signaling molecules, putative immunometabolic interaction networks can be inferred. For instance, regions enriched for PD-1-expressing T cells adjacent to PD-L1-expressing myeloid cells suggest the presence of localized immunosuppressive hubs within the TME [140]. The presence or absence of these spatial immune niches has been shown to be correlated with clinical outcomes, providing clinicians with improved prognostic insight and informing therapeutic strategies [141]. Collectively, spatial biology approaches preserve both cellular identity and tissue context, uncovering immune niches and functional gradients that are obscured by dissociative single-cell methods. By integrating location with cell state, these technologies offer transformative insight into immune reprogramming and disease progression in GBM [79]. Figure 1 synthesizes the multidimensional architecture of the glioblastoma tumor microenvironment, highlighting the cellular, spatial, and metabolic networks that govern immune suppression and define actionable vulnerabilities for next-generation immunotherapeutic strategies.

## 5. Emerging Translational Insights

Advances in single-cell and spatial imaging technologies have revealed fibroblast-like stromal populations in the glioblastoma (GBM) microenvironment, which was previously considered largely fibroblast-free [142]. These cancer-associated fibroblast (CAF)-like cells can arise from multiple progenitors, including pericytes, vascular smooth muscle-like cells, mesenchymal tumor cells, and reactive astrocytes [143,144,145]. In other cancers, TGF-β, PDGF, and hypoxia drive pericytes to adopt CAF-like programs characterized by upregulation of extracellular matrix remodeling genes that are also upregulated in GBM CAF-like cells such as COL1A1/2 and FN1 [146,147,148,149]. CAF-like cells are enriched in mesenchymal GBM and localize to perivascular and hypoxic regions of the tumor microenvironment, where they may restrict T-cell trafficking into the tumor parenchyma [143]. These cells secrete TGF-β, IL-6, and IL-8 to promote immunosuppression by inducing T-cell dysfunction and exhaustion while recruiting and skewing myeloid populations toward pro-tumor phenotypes [88,150]. By limiting T-cell infiltration and effector function, CAF-like cells may contribute to the failure of immune checkpoint blockade in GBM. Thus, reprogramming CAF-like cells and targeting CAF-associated pathways are exciting new therapeutic avenues for improving the efficacy of immunotherapy.

### 5.1. Next-Generation and Novel Immunotherapeutic Strategies

Immunotherapy has thus far failed to confer meaningful clinical benefit in GBM. This disparity reflects fundamental immunological barriers intrinsic to solid tumors of the central nervous system that catalyzed a new generation of therapeutic strategies for GBM, including combinatorial regimens, modulation of myeloid and stromal compartments, metabolic and cytokine rewiring of T cells, neutrophils, macrophages and advanced CAR engineering, and novel delivery technologies to enhance intracranial penetration and spatial control [147,151,152,153,154,155,156,157,158].

### 5.2. Combinatorial Approaches

Immune checkpoint blockades (ICB) reinvigorate antitumor T-cell response by alleviating immune suppression. However, GBM remains largely resistant to ICB [159,160] owing to a common lack of pre-existing antitumor immunity [161,162]. Against this backdrop, combining ICB with cancer vaccines has emerged as a rational improvement approach. In principle, cancer vaccines provide the missing upstream actions required for checkpoint blockade to exert downstream efficacy by expanding tumor-specific T-cell repertoires, enhancing antigen presentation, and increasing immune infiltration into otherwise “cold” tumors.

Early clinical trials with this strategy have been modest but encouraging. A phase I/II study combining a DNA vaccine with the PD-1 inhibitor cemiplimab alongside standard chemoradiation in newly diagnosed GBM reported a median overall survival of 32.5 months in MGMT-methylated patients [163]. Similarly, a randomized phase IIb trial evaluating neoadjuvant PD-1 inhibitor pembrolizumab combined with a glioma stem cell lysate-loaded dendritic cell vaccine in recurrent GBM demonstrated an improvement in median overall survival (22.7 months versus 8.2 months with pembrolizumab alone), although there was no difference in progression-free survival [164].

Multiple ongoing clinical trials are also testing different vaccine-based combinations with ICB for GBM. One example is a phase 1 clinical trial of a personalized neoantigen peptide vaccine, in which synthetic tumor-specific peptides that prime antigen-specific T-cell responses are administered in combination with radiation therapy plus pembrolizumab and TMZ for newly diagnosed GBM (NCT02287428). Another approach is combining cytomegalovirus (CMV)-targeted immunotherapies, which leverage the selective expression of CMV antigens within tumor cells, to improve ICB by counteracting adaptive immune resistance within the immunosuppressive GBM TME (NCT06157541). Beyond tumor-specific vaccination, recent work has revealed a more unexpected avenue for immune sensitization in which effective immune priming may not require tumor-specific antigens at all. Notably, SARS-CoV-2 mRNA vaccines have been shown to sensitize otherwise resistant tumors to ICB in non-small cell lung cancer and melanoma by inducing a transient, type I interferon-driven innate immune response that activates antigen-presenting cells, primes tumor-reactive CD8^+^ T cells, and promotes adaptive immune infiltration [165]. Substitution of spike mRNA with glioma-associated CMV antigens did not further enhance anti-tumor efficacy, implying that innate immune sensing of the mRNA–lipid nanoparticle platform itself may be the dominant driver of immune reprogramming.

Conventional therapies such as radiotherapy can also serve as a potent means to prime and modulate the TME in GBM. Beyond direct cytotoxicity, early preclinical studies indicate that radiation can increase neoantigen presentation and MHC Class I expression, enhancing CD8^+^ T-cell activation and infiltration [166,167,168,169]. Radiation-induced increases in vascular permeability further enhance immune infiltration [166,170,171], while the induction of cytokines can modulate antitumor immunity in either a stimulatory or inhibitory manner [168]. Notably, these immunomodulatory effects are often most pronounced with limited radiation fractions (~10 Gy) [168,172]. In contrast, chemotherapy can induce profound lymphopenia, impairing subsequent immunotherapy efficiency [99,173,174,175]. These mechanistic insights provide a rationale for integrating radiotherapy into combinatorial approaches that sequence immune priming, checkpoint blockade, and conventional therapy, for instance prior to anti-PD-1 therapy, and followed by surgical resection and adjuvant chemoradiotherapy [168,172,173,176].

A natural extension of these strategies has been to explore innate immune modulators such as oncolytic viral therapy as adjuvants to engineered CAR T-cell therapies, with the goal of reshaping the tumor microenvironment to support CAR T-cell expansion and persistence. Through selective infection and lysis of tumor cells, oncolytic viral therapy is proposed to synergize with CAR T-cell therapy by inflaming the TME, promoting tumor antigen release, enhancing antigen presentation, and inducing chemokines that facilitate CAR T-cell trafficking and local activation [177]. Engineered oncolytic platforms can further amplify this synergy by delivering CAR target antigens or immunostimulatory signals directly within the tumor, spatially restricting CAR engagement and reducing antigen escape while minimizing off-tumor toxicity.

While there are currently no trials specifically on GBM, many clinical trials combining oncolytic virus and CAR T are underway for many end-stage solid tumors (NCT03740256, NCT05057715). However, preclinical experiments have revealed important biological constraints for this approach. Tonne and colleagues investigated the combination of an oncolytic foamy virus with CD19-targeted CAR T cells in which they engineered tumor cells to transiently express the CAR target during viral infection [178]. Unexpectedly, this strategy failed to improve outcomes in subcutaneous murine models of high-grade glioma; indeed, the greatest antitumor efficacy was achieved with high-dose oncolytic virus alone [178,179]. Mechanistically, this paradox appears to stem from an oncolytic virus-induced type I interferon signaling, particularly IFN-β. While type I interferons enhance innate immune activation and tumor immunogenicity, they can simultaneously drive CAR T-cell attrition through apoptosis, tonic CAR hyperactivation, and upregulation of inhibitory receptors such as PD-1, LAG-3, and TIM-3, ultimately undermining persistence and effector function. As demonstrated by Evgin and colleagues, this interferon-mediated negative feedback can outweigh the immunostimulatory benefits of viral oncolysis, highlighting that OV–CAR T combinations are unlikely to succeed unless virus-induced type I interferon toxicity to engineered T cells is specifically mitigated [179]. Going forward, oncolytic virotherapy is likely to function as an effective adjuvant to CAR T-cell therapy only if its beneficial tumor-inflaming effects can be uncoupled from IFN-β inhibitory signals that limit T-cell persistence and function.

In parallel with advances in cellular and biological therapy, minimally invasive physical interventions have emerged as complementary strategies to reshaping TME. One such strategy is the integration of immunotherapy with laser interstitial thermal therapy (LITT), which has the potential to convert immunologically “cold” tumors into more permissive targets for immunotherapy. LITT induces focal hyperthermia that achieves selective tumor killing while largely preserving surrounding brain tissue [180]. MRI-guided LITT has demonstrated an acceptable safety profile and clinical utility in the management of both newly diagnosed and recurrent GBM [181,182,183]. Most importantly, LITT appears to also exert biologically meaningful immunomodulatory effects. Post-LITT tumor specimens show increased infiltration of CD8^+^ T cells and activated macrophages, alongside upregulation of PD-L1, suggesting induction of a transient inflammatory state that may render tumors more responsive to ICB. Consistent with this rationale, a phase I/II study combining LITT with the anti-PD-1 antibody pembrolizumab in recurrent GBM reported improved progression-free (median PFS 10.5 months vs. 2.1 months) and overall survival (median OS 11.4 months vs. 5.2 months) compared with pembrolizumab alone (NCT03277638) [184]. Ongoing studies, including NCT03277638, will be essential to define how the timing, extent, and spatial precision of LITT can be harnessed to improve future immunotherapy. Table 2 outlines emerging biomarker classes for precision immunotherapy stratification, highlighting how interferon competence, myeloid functional states, exhaustion phenotypes, spatial architecture, and immunometabolic signatures can be operationalized for patient selection and response monitoring.

### 5.3. Engineering Solutions

Building on advances in immune priming and TME reprogramming, engineering-focused solutions on next-generation “armored” CAR T cells have emerged to endow CAR T cells with autonomous ability to survive, function, and persist inside the immunosuppressive and metabolically hostile landscape of GBM’s TME. Unlike first-generation CAR T cells, which rely heavily on external immune support, armored CAR T cells are genetically modified to deliver intrinsic advantages such as cytokine secretion, resistance to inhibitory signaling, enhanced chemotaxis, and sustained co-stimulation. Several such approaches have now advanced into early-phase clinical trials.

Armored CAR T cells carrying cytokine payloads represent a first generation of engineering solutions designed to overcome the trafficking and functional barriers imposed by the GBM TME. One example is an ex vivo expanded autologous CXCR2 CD70 CAR (8R-70CAR) T trial (NCT06946680), which pairs a CAR T targeting CD70+, a surface antigen enriched in glioma stem-like cells and recurrent GBM, with the IL8 chemokine receptor CXCR2. As GBM secretes high levels of IL-8 to recruit suppressive myeloid cells, CXCR2 expression enables CAR T cells to hijack this endogenous chemokine gradient, thereby enhancing CAR T cell chemotaxis and tumor infiltration [185].

In addition to secreting cytokine payloads, CAR T cells can also be engineered to actively resist immunosuppressive cytokine signaling secreted by GBM such as TGF-β and IL-10. One prominent strategy involves armoring CAR T cells with dominant-negative receptors, which are synthetic non-signaling version of these immunosuppressive cytokine receptors that act as ligand sinks that sequester inhibitory cytokines without transducing downstream suppressive signals. In preclinical models, a bispecific EGFR/IL13Rα2 CAR T cell armed with a dominant-negative TGF-β receptor II (dnTGFβRII) has demonstrated particular promise [186]. When combined with bicistronic EGFR–IL13Rα2 targeting, dnTGFβRII armoring significantly enhanced CAR T-cell proliferation, effector function, and antitumor efficacy, while simultaneously improving safety in murine GBM models [186].

More elaborate fourth-generation “multi-armored” CAR T designs are now being explored. Such a construct incorporates an IL13Rα2-targeting CAR together with a TGF-β-pathway blocking antibody, providing sustained resistance to TGF-β-mediated suppression while extending CAR T-cell survival and proliferative capacity in the absence of exogenous cytokine support. Importantly, this platform also integrates a truncated HER2 safety switch, enabling rapid pharmacologic elimination of CAR T cells in the event of uncontrolled expansion or toxicity, thereby enhancing translational safety [187].

### 5.4. Metabolic Immunotherapy

Metabolic reprogramming within the tumor microenvironment actively shapes immune cell fate, creating conditions that suppress effector immunity while reinforcing tolerance, and may be one mechanism by which GBM has resisted current clinical trials. Among these pathways, dysregulated tryptophan (trp) and lactate metabolism have attracted particular attention as tractable drivers of immunosuppression.

Tumors can hijack Trp catabolism to create an immunosuppressive TME to support their growth and survival through the indoleamine 2,3-dioxygenase 1 (IDO1)–kynurenine–aryl hydrocarbon receptor (AHR) axis, which accounts for the majority of tryptophan flux in inflammatory settings [188]. The IDO1–Kyn–AHR axis is an immunoregulatory negative feedback loop in which inflammatory cues induce IDO1 to catabolize Trp into Kyn, and Kyn activates AHR on immune cells to reinforce tolerogenic gene programs (e.g., increase Treg/tolerogenic DC features, decrease pro-inflammatory mediators), thereby damping inflammation [189]. While some immune and epithelial cells only express IDO1 and TDO2 in response to inflammation signals, many tumor cells, including GBM, express IDO1 and TDO2 constitutively to suppress host immune killing and promote tumor immune invasion [190]. These programs promote regulatory T-cell differentiation, impair dendritic cell immunogenicity, and suppress pro-inflammatory cytokine production, thereby limiting effective anti-tumor immunity.

This mechanistic insight has motivated multiple clinical efforts to disrupt tryptophan metabolism in GBM. Indoximod, an IDO pathway inhibitor, is currently being evaluated in combination with temozolomide (NCT02502708), radiotherapy (NCT04049669), ICB (NCT04047706, NCT03707457), and anti-angiogenic therapy (NCT02052648) across several trials. In parallel, next-generation proteolysis-targeting chimeras (PROTACs) directed against IDO1 couple high-affinity IDO1 binding to E3 ligase recruitment, triggering targeted degradation of IDO1 via the ubiquitin–proteasome system and achieving improved tissue penetration and markedly enhanced potency in preclinical orthotopic GBM models [191,192].

In addition to tryptophan metabolism, lactate accumulation represents a second metabolic barrier to effective immunity in GBM. Like many solid tumors, GBM preferentially relies on aerobic glycolysis, which diverts metabolic flux away from oxidative phosphorylation toward lactate production, leading to extracellular acidification, and promotes immunosuppression by driving macrophage polarization toward an M2-like state [193,194,195]. Moreover, lactate signaling has been linked to resistance to PD-1 blockade through increased PD-1 expression on regulatory T cells, further constraining anti-tumor responses [196].

Therapeutic strategies targeting lactate metabolism are beginning to emerge. One biomimetic nanoparticle platform converts tumor-derived lactate into pyruvate and hydrogen peroxide, simultaneously disrupting epigenetic regulation, inducing cell-cycle arrest, and enabling chemiexcited photodynamic cytotoxicity. In patient-derived xenograft models of GBM, this approach successfully crossed the blood–brain barrier and significantly extended survival relative to controls [197]. Although still preclinical, such strategies highlight the potential of metabolic interventions to reshape the immune landscape rather than merely target tumor cell viability.

## 6. Frontiers in Delivery

The therapeutic effects of next-generation immunotherapies in GBM are ultimately constrained not only by biology but also by delivery owing to the inherent location of the tumor within the immune-privileged blood–brain barrier [70]. Recent innovations in the realm of immunotherapeutic delivery include focused ultrasound (FUS), convection-enhanced delivery (CED), and biomaterial scaffolds.

Ultrasound is a form of mechanical energy that can be transmitted efficiently through deep tissue. FUS can enhance therapeutic delivery across the BBB by delivering pulsed, low-intensity ultrasound focusing on specific locations within the brain along with intravenous microbubbles. Mechanical energy from FUS allows microbubbles to expand and contract inside the brain capillaries and permeate the BBB [198]. In recent years, FUS applications in drug delivery have expanded from chemotherapies to biological drugs, including genes, viruses, antibodies, and cell-based therapies [199,200,201,202,203]. FUS has been used to enhance delivery of CAR T cells [204] and of IL-12 [205] to increase tumor infiltration in preclinical models. In addition, FUS also has an additional effect in increasing immunogenicity of tumors when delivered in high intensity. Mechanical energy of FUS causes tumor cell lysis and releases tumor-associated antigens and promotes antigen presentation [198]. Unfortunately, such immunomodulatory effects of FUS have not yet been observed in human trials despite BBB permeability being observed in one preclinical study [206]. That said, FUS has been shown to enhance gene therapies for other neurological disorders [207], such as traumatic brain injuries, epilepsy, stroke, and cerebrovascular diseases, suggesting that FUS has therapeutic potential in humans [205,207,208,209].

Convection-enhanced delivery (CED) is another method that aims to bypass the BBB by using a stereotactically implanted catheter and syringe pump to create a pressure gradient that delivers immunotherapeutic drugs directly to the brain parenchyma [210]. Many early trials, mostly phase I and II, have investigated the safety and efficacy of CED in delivering antibodies and oncolytic viruses (NCT04160494, NCT04479241, NCT01491893, NCT00076986). However, all early trials and a phase III trial [211] produced no objective radiographic responses. A more recently developed approach, electrokinetic CED, uses an external electric field to infuse drugs to the parenchyma, without the need for a pressure gradient from a syringe pump, and is currently in development in preclinical studies [212].

One other challenge in the delivery of immunotherapy, especially for adoptive cell transfer therapies such as CAR T and TCR T, is the stability and viability of therapeutic cells, often due to insufficient cytokines and growth factors in vivo. Li and colleagues showed that an implantable hydrogel–fiber device placed in the GBM resection cavity prevented tumor recurrence in 40% of mice and significantly prolonged survival by restoring effective local antitumor immunity [213]. Mechanistically, the hydrogel neutralizes the acidic postoperative tumor microenvironment and enables spatiotemporally controlled release of CXCL10 and a PD-L1 inhibitor, thereby recruiting cytotoxic T cells, preventing their functional exhaustion, and synergistically amplifying immune checkpoint blockade efficacy at the tumor site [213]. Table 3 presents a next-generation combination immunotherapy design framework that maps specific tumor microenvironment constraints to rational therapeutic layers and biomarker-guided trial design strategies.

## 7. Future Directions and Opportunities

The landscape of glioblastoma (GBM) treatment is considerably impacted by suboptimal clinical trial design, such as lack of adequate control arms and single-arm designs in phase II trials [214,215], stringent eligibility criteria [216,217], and limited incorporation of prognostic and predictive biomarkers [218,219,220,221]. Implementing biomarker-driven adaptive trials has the potential to enhance therapeutic precision through patient stratification and personalization. This can be achieved by integrating advanced methodologies such as single-cell and spatial immunophenotyping into clinical trial frameworks, which allows incorporation of insights from intra-tumoral heterogeneity and immune microenvironments [222].

In parallel, the integration of artificial intelligence with cancer neuroscience is likely to drive the development of next-generation immunotherapies for GBM. This prediction is instantiated by the rising ability of AI systems to facilitate the fusion of multiple data types across imaging, spatial omics, and clinical data sets to identify the immunological niches and resistance profiles of the tumor microenvironment [223,224,225,226]. AI systems could also potentially facilitate the development of predictive models that support dynamic patient stratification and response monitoring, as well as combination therapy development to more efficiently target the GBM immunomicroenvironment [226,227,228].

As therapeutic strategies evolve, precision immunotherapy will rely heavily on patient stratification based on factors such as methylation class [229,230,231], immune signatures [232], or spatial phenotypes [233,234]. Understanding the immune landscape in GBM can direct novel therapies, such as the combination of immune checkpoint inhibitors and tumor-specific vaccines, tailored to individual molecular profiles that may yield better outcomes in certain patient subgroups [235]. Although combination therapies involving anti-PD1/anti-CTLA4 have shown limited efficacy [236,237], lineage-specific checkpoint inhibition strategies targeting LAG3, TIM3, and IDO1 are being explored. Furthermore, non-invasive liquid biopsies are emerging as valuable tools for monitoring treatment efficacy and tumor progression, enabling timely treatment modifications [238].

In addition, insights from meningiomas suggest that PIK3CA pathway activation and hormonal receptor signaling can cooperate to facilitate tumor growth through convergent metabolic, proliferative, and immune-modulatory effects, and such convergent effects might also be seen in the molecular subgroups of GBMs through PI3K-mediated endocrine signaling pathways. Translationally, such findings suggest that it might be worthwhile to explore the efficacy of combined therapeutic strategies targeting GBMs through simultaneous inhibition of the PI3K/AKT/mTOR pathway and modulation of steroid hormone and nuclear receptor signaling to overcome adaptive resistance and modulate the immune microenvironment [239,240,241,242,243].

Harnessing multi-omics data, including genomics, proteomics, metabolomics, and advanced imaging modalities, presents a frontier in GBM research, enabling biomarker identification and treatment strategy formulation [10,244]. By identifying specific perturbed metabolic pathways and molecular fingerprints associated with different GBM subtypes, better prognosis could be attained [230]. As an example, early identification of GBM subtypes associated with improved survival can optimize enrollment in clinical trials and drive personalized therapeutic interventions. Moreover, advances in machine learning can be applied to enhance the interpretation of multi-omics data, improving predictions of treatment success [227,244,245,246].

Artificial intelligence has the potential to revolutionize several aspects of GBM, from early diagnosis to treatment planning and prognostication [247]. AI-driven models can enhance clinical trial design by better correlation of patient characteristics with treatment response. Technical advancements in AI-assisted intraoperative imaging and mass spectrometry can support real-time surgical decision-making, improving the effectiveness of surgical resections [227]. An interdisciplinary approach that combines molecular oncology, neurosurgery, and data science will likely lead to a robust treatment strategy that addresses the complex nature of GBM.

To realize the full potential of immunotherapy in GBM, therapeutic interventions must be multimodal, context-specific, and dynamically adaptive. Given the high degree of heterogeneity in GBM, a one-size-fits-all approach is unlikely to succeed. Instead, combining various therapeutic modalities while adapting strategies based on molecular and immunological feedback will enhance treatment efficacy. Leveraging real-world data from biobanks and liquid biopsies using cutting-edge machine learning and artificial intelligence tools will enable refining therapeutic options and ensuring each patient receives the optimal treatment based on their unique tumor biology.

## Figures and Tables

**Table 1:** Mechanistic Axes of Immunotherapy Resistance in Glioblastoma.

Resistance Axis	Core Mechanism	Cellular Drivers	Functional Consequence	Biomarker Readouts	Therapeutic Leverage Points
Interferon signaling dysfunction	Impaired IFN sensing or downstream transcriptional response	Tumor cells, myeloid cells	Reduced antigen presentation, blunted checkpoint response, immune exclusion	IFN response gene sets, STAT1/IRF1 programs, MHC-I expression	IFN pathway restoration, STING agonists, IFN-priming combinations
Myeloid antigen presentation failure	TAM/TAN skewing toward suppressive, low-APC phenotypes	TAMs, MDSCs, neutrophil subsets	Poor T-cell priming, T-cell dysfunction despite infiltration	HLA-II, CD74, CD83, costimulatory ligand expression	Myeloid reprogramming, CSF1R modulation, APC-state induction
T-cell exhaustion programming	Chronic antigen exposure + suppressive niche signaling	CD8 TILs, CAR-T cells	Reduced cytotoxicity and proliferative capacity	PD-1, LAG3, CD39, TOX, exhaustion transcriptional modules	Multi-checkpoint blockade, metabolic rescue, antigen load control
Metabolic resource competition	Nutrient depletion and suppressive metabolite accumulation	Tumor cells, myeloid cells	T-cell metabolic insufficiency and effector collapse	Glycolysis/OXPHOS gene sets, lactate transporters, adenosine pathway markers	Metabolic checkpoint blockade, adenosine receptor inhibition
Spatial immune exclusion	Physical and signaling barriers to immune penetration	Tumor stroma, vascular niche	Immune cells restricted to perivascular or margin niches	Spatial immune mapping, immune distance metrics	Vascular normalization, matrix remodeling
Intratumoral immune heterogeneity	Divergent immune niches within same tumor	Mixed immune populations	Mixed treatment response, partial resistance	Single-cell + spatial immune profiling	Region-aware therapy design, adaptive dosing
Antigen presentation instability	Dynamic antigen loss or processing defects	Tumor cells	Immune escape under therapy	Neoantigen burden, β2M loss, MHC downregulation	Multi-antigen targeting, epitope spreading strategies

**Table 2:** Emerging Biomarker Classes for Precision GBM Immunotherapy.

Biomarker Class	What It Captures	Measurement Platform	Clinical Utility	Strengths	Limitations
Interferon competence signatures	Tumor immune responsiveness potential	Bulk RNA, single-cell RNA	Predict checkpoint sensitivity	Mechanistically anchored	Dynamic and therapy-dependent
Myeloid functional states	APC vs. suppressive myeloid balance	Single-cell RNA, spatial proteomics	Stratify myeloid-targeted combinations	Directly links to T-cell priming	Requires high-dimensional assays
T-cell clonality and expansion	Antigen-driven immune engagement	TCR sequencing	Response likelihood, immune activation tracking	Quantitative and reproducible	Does not guarantee functionality
Exhaustion marker co-expression	Functional T-cell impairment	Flow, CyTOF, spatial IF	Functional immune status	Translationally accessible	Marker expression
Spatial immune architecture	Immune–tumor proximity and niche structure	Spatial transcriptomics, multiplex IF	Predict response niches and escape zones	Preserves context	Cost and analytic complexity
Immunometabolic signatures	Metabolic suppression and competition	Single-cell RNA, metabolomics	Combination design (metabolic + immune)	Mechanism-driven	Platform variability
Adenosine pathway activation	Immunosuppressive metabolite signaling	Flow, transcriptomics	Target selection for metabolic checkpoint therapy	Drug-targetable axis	Context-dependent
Dynamic pharmacodynamic markers	Early therapy response signals	Serial biopsy, liquid biomarkers	Adaptive trial decision-making	Enables response-guided design	Requires longitudinal sampling

**Table 3:** Next-Generation Combination Immunotherapy Design Framework for GBM.

Therapeutic Layer	Targeted Constraint	Example Strategy Type	Rationale	Required Biomarker Support	Trial Design Implication
Checkpoint axis	T-cell inhibitory signaling	Dual/triple checkpoint blockade	Releases inhibitory signaling	Exhaustion marker profiling	Enrich for exhaustion-high tumors
Interferon axis	IFN signaling insufficiency	IFN priming + checkpoint	Restores immune activation competence	IFN gene signature	Stratify by IFN competence
Myeloid axis	APC failure/suppressive TAMs	Myeloid reprogramming agents	Improves antigen presentation	Myeloid state markers	Myeloid-state stratified cohorts
Metabolic axis	Nutrient and metabolite suppression	Adenosine or metabolic blockade	Restores T-cell energetics	Metabolic signatures	Combine with T-cell therapies
Spatial axis	Immune exclusion	Vascular or matrix modulators	Improves immune penetration	Spatial immune maps	Region-aware endpoints
Cellular therapy axis	Effector insufficiency	CAR-T/engineered T cells	Direct cytotoxic replacement	Antigen density + TME profile	Combine with TME modulators
Antigen axis	Target escape	Multi-antigen targeting	Reduces escape probability	Antigen heterogeneity mapping	Multi-target enrollment
Adaptive monitoring axis	Dynamic resistance	Biomarker-adaptive dosing	Responds to evolving biology	Serial immune profiling	Adaptive trial platforms
Radiotherapy axis	Low antigenicity and immune exclusion	Hypofractionated RT	Increases neoantigen presentation, MHC-I expression, vascular permeability	Neo-antigen burden, DNA damage response	Neoadjuvant RT window, 10 Gy priming doses

## Data Availability

No new data were created or analyzed in this study.
